# Preliminary Findings of Platelet-Rich Plasma-Induced Ameliorative Effect on Polycystic Ovarian Syndrome

**DOI:** 10.22074/cellj.2019.5952.

**Published:** 2019-06-15

**Authors:** Samira Seyyed Anvari, Gholamreza Dehgan, Mazdak Razi

**Affiliations:** 1 Department of Biology, Collage of Post Graduate, Ahar Islamic Azad University, Ahar, Iran; 2 Department of Biochemistry, Faculty of Natural Science, University of Tabriz, Tabriz, Iran; 3 Department of Basic Science, Faculty of Veterinary Medicine, Urmia University, Urmia, Iran

**Keywords:** Folliculogenesis, Oxidative Stress, Platelet-Rich Plasma, Polycystic Ovarian Syndrome, Rat

## Abstract

**Objective:**

Polycystic ovarian syndrome (PCOS) is characterized by hormonal imbalance, oxidative stress and chronic anovulation. The present study was designed to assess ameliorative effect of auto-locating platelet-rich plasma (PRP), as a novel method, for inhibiting PCOS-induced pathogenesis in experimentally-induced hyperandrogenic PCOS.

**Materials and Methods:**

In this experimental study, 30 immature (21 days old) female rats were assigned into five groups, including control (sampled after 30 days with no treatment), 15 and 30 days PCOS-sole-induced as well as 15 and 30 days PRP auto-located PCOS-induced groups. Serum levels of estrogen, progesterone, androstenedione, testosterone, follicle stimulating hormone (FSH), luteinizing hormone (LH), ovarian total antioxidant capacity (TAC), malondialdehyde (MDA), glutathione peroxidase (GSH-px) and superoxide dismutase (SOD) were evaluated. Expression of estrogen receptor α (Erα), β (Erβ) and c-Myc were assessed. Finally, the numbers of intact follicles per ovary and mRNA damage ratio were analyzed.

**Results:**

PRP groups significantly (P<0.05) decreased serum levels of FSH, LH, testosterone and androstenedione and remarkably (P<0.05) increased estrogen and progesterone syntheses versus PCOS-sole groups. The PRP auto-located animals exhibited increased TAC, GSH-px and SOD levels, while they showed diminished MDA content (P<0.05) versus PCOS-sole groups. The PRP auto-located groups exhibited an elevated expression of Erα and Erβ versus PCOS-sole groups. Moreover, PRP groups significantly (P<0.05) decreased c-Myc expression and mRNA damage compared to PCOS-sole groups, and remarkably improved follicular growth.

**Conclusion:**

PRP is able to regulate hormonal interaction, improve the ovarian antioxidant potential as well as folliculogenesis and its auto-location could be considered as a novel method to prevent/ameliorate PCOS-induced pathogenesis.

## Introduction

Polycystic ovarian syndrome (PCOS) is an exceptionally common disorder, which is widely observed in premenopausal women. It is characterized by an increased serum level of androgens (hyperandrogenism), chronic anovulation and presence of the polycystic ovarian morphology (1). According to the Rotterdam consensus in 2003, chronic anovulation or oligomenorrhea, clinical or biochemical hyperandrogenism, and polycystic ovarian morphology are declared as main criteria for PCOS (2). Among the different mentioned phenotypes, ovarian hyperandrogenism has gained higher attenuations. Indeed, in PCOS, an intrinsic steroidogenic defect of theca cells results in ovarian hyperandrogenism. Accordingly, increased LH and enhanced insulin levels amplify inherent impairment of steroidogenesis in theca cells (3). In addition to hyperandrogenism symptoms, follicle stimulating (FSH) and luteinizing (LH) hormones up- regulation, as well as estrogen and progesterone reduction levels have been reported in PCOS patients (3, 4). Estrogen interacts with two distinct estrogen receptors (ERs), namely ERa and ERß (5), both of which regulate variety of genes expression, leading to cellular proliferation and differentiation in both male and female gonads (6). In rodents, *Erα* is expressed exclusively in theca cells, whereas *Erß* is expressed especially in granulosa cells (GCs) (7). Several evidences, including failed follicular maturation, anovulation and hemorrhagic cysts formation are reported for Erα knockout (aERKO) mice ovaries (8, 9). The *Erß*-related phenotypes are partially different from those related to Erα. Actually, Erß knockout (ßERKO) mice ovaries appear normal, exhibiting follicles at all stages of development. Meanwhile, these mice represent fewer corpora lutea, resulting in mild subfertility problems. Moreover, failed response to exogenous gonadotropins as well as a severe deficiency in response to the LH/human chorionic gonadotropin (hCG) ovulatory stimulus have been reported in ßERKO mice ovaries (5). 

In addition to estrogen and ERs, the proto-oncogene cellular myc (*c- Myc*), as a transcription factor, participates in cellular proliferation pathway (10). Although c-Myc protein has been illustrated to induce both growth and oncogenic properties, very early studies have shown its pro-apoptotic characteristic in ovarian tissue. c-Myc is expressed in GCs at all stages of follicular development and in oocyte of primordial follicles, suggesting its role in remodeling the ovarian local tissue following atresia and luteolysis (11). Meanwhile, the massive expression of c-Myc protein in GCs and theca interna of atretic follicles, as well as peripheral theca lutein cells implies the pro-apoptotic characteristic of c-Myc in ovaries (12). 

According to the previous reports, PCOS is frequently associated with oxidative stress. Various investigations have shown remarkable enhancement in circulating malondialdehyde (MDA) as well as significant reduction in serum superoxide dismutase (SOD), and glutathione peroxidase (GSH-px) of patients with PCOS (13, 14). Indeed, there is a positive correlation between obesity, insulin resistance, hyperandrogenemia, chronic inflammation and oxidative stress in PCOS ovaries (15). Therefore, the impressively-induced oxidative stress is considered as a potential inducer of PCOS-related pathogenesis (13).

Platelet-rich plasma (PRP) or autologous platelet gel, has gained high attentions in musculoskeletal medicine, hemostasis and wound healing, inhibiting immune reactions, aesthetic plastic surgery (16), spinal fusion (17) and heart bypass surgery (18), in addition to treatment of chronic skin and soft-tissue ulcers (19). a-granules of platelets are comprised of numerous proteins, including platelet-derived growth factor (PDGF), transforming growth factor (TGF)-ß, platelet factor 4 (PF4), interleukin (IL)-1, platelet-derived angiogenesis factor (PDAF), vascular endothelial growth factor (VEGF), epidermal growth factor (EGF), platelet-derived endothelial growth factor (PDEGF), epithelial cell growth factor (ECGF) insulin-like growth factor (IGF), osteocalcin, osteonectin, fibrinogen, vitronectin, fibronectin, and thrombospondin (TSP)-1 (16). Further studies have shown promoting effect of PRP in different therapeutic innervations (20).

Minding the essential role of growth hormones in both early and late folliculogenesis and in initiating oocyte growth, as well as cell proliferation and inhibiting apoptosis (especially at later stages of development), this question arises what the possible effect of PRP- related growth factors on different molecular elements is, in PCOS-induced ovaries. Therefore, here we aimed to uncover the possible ameliorative effect of PRP on hyperandrogenic PCOS-induced derangements in ovarian tissue. The possible PRP-related ameliorative effects were assessed in five well-established categories, including: i. Alterations at gonadotropins, androgens, estrogen and progesterone levels, ii. Changes in expression of Erα and Erß (as important receptors participating in folliculogenesis), iii. Alteration in c-Myc expression (as important proto-oncogene involved in cell proliferation/ apoptosis), iv. Ovarian antioxidant status, and finally v. Follicular atresia and/or growth ratio.

## Materials and Methods

### Chemicals and materials

Specific commercial kits were purchased for analysis of rat testosterone (Mybiosource, USA), androstenedione (Mybiosource, USA), estrogen (Bio Vender, Czech Republic), progesterone (Crystal Chem, USA), LH (Mybiosource, USA), FSH (Bio Vender, Japan). Primary antibodies were provided for Erα, Erß and c-Myc (Rabbit- Antimouse Erα, Erß and c-Myc; Biocare, USA). Commercial kits for SOD and GSH-px were obtained from RANDOX reagents company (Germany). All other chemical agents were commercial products of analytical grade. 

### Animals, PCOS induction and experimental design 

**Fig.1:**
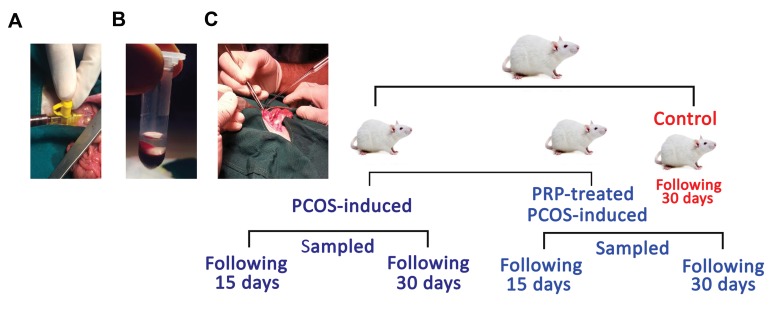
Summarized schematic diagram for animals platelet-rich plasma (PRP) preparation, activation, auto-location and animal grouping of the study. A. Blood sampling from caudal vena cava, **B.** PRP preparation, and C. PRP auto-location.

The current experimental study was performed on animal models. To conduct it, 30 immature (21 days old) female Sprague-Dawley rats were assigned into five groups (six rats in each group), including control (sampled after 30 days), PCOS-induced (sampled 15 and 30 days of post PCOS induction) and PRP auto-located PCOS- induced (sampled 15 and 30 days of post PCOS induction) groups. The animals were given ad libitum access to food and water, kept at room temperature (21-23oC) on a 12:12 light:dark cycle. The hyperandrogenic PCOS- like condition was induced based on the previous study by Honnma et al. (21). Briefly, dehydroepiandrosterone (DHEA, 6 mg/100 g body weight/0.2 ml sesame oil) was subcutaneously injected to 22 days old rats, every evening for 15 days. The animals in the control group were received 0.2 ml sesame oil every evening for the corresponding length of time. Extra cares were taken and no inflammatory reaction was observed at the injected site, during the trial (Fig .1). All experimental protocols were approved and monitored by the Ethical Committee in Animal Experimentation of Urmia University (Urmia, Iran).

### Platelet-rich plasma preparation, activation and count 

To perform the experimental procedures and PRP preparation, the animals were anesthetized through intraperitoneal injection of xylazine (6 mg/kg, Trittau, Germany) and ketamine (70 mg/kg, Alfason-Woerden, Netherland). Next, the cannulation of caudal vena cava was submitted. 5 ml disposable syringe containing 0.35 ml of 10% sodium citrate was used to collect 3.15 ml PRP of each animal. The blood samples were kept in 5 ml sterile silicone vacuum tubes. In order to replace the same amount of blood, sterile saline was immediately injected. PRP preparation was carried out based on the proposed protocol by Messora et al. (22). Briefly, the collected blood samples were firstly centrifuged (Beckman J-6M, UK) at 160 rpm, 22°C for 20 minutes. Then, red blood cell component (lower fraction) and serum component, as an upper straw-yellow turbid fraction, were observed. Thereafter, a point was marked at 1.4 mm below the line dividing two fractions. All contents above the marked point were pipetted and transferred to another 5 ml vacuum tube. The sample was then centrifuged at 400 rpm, for 15 minutes, resulting in two components, including platelet- poor plasma (PPP) and PRP in the bellow part (Fig .1A, B). Next, similar amounts of PRP and PPP (0.35 ml) were pipetted and transferred to different sterile dappen dishes. After that, they were activated by adding 0.05 ml of 10% calcium chloride solution to each 1 ml of PRP or PPP. Finally, the platelets were manually counted (8.08 ± 3.24×106/µl) using the Neubauer chamber, through Olympus optical microscope (CH-2, Japan), at ×40 magnification objective lens. 

### Auto-location of platelet-rich plasma

Following PCOS induction, PRPs were collected and activated as previously described and subsequently 1.00×10^6^/µl PRPs were auto-located from each animal into the mesovarian enclosed to ovaries (Fig .1C).

### Histological analyses

At the end of experiment, light anesthesia was induced to animals using 5% ketamine (40 mg/kg) in addition to 2% xylazine (5 mg/kg), intraperitoneally and then euthanized by especial CO2 device (ADACO, Iran). Next, the ovarian tissues were dissected out and fixed in 10% formalin for 72 hours. Routine sample processing was performed using ascending alcohol and the samples were then embedded in paraffin. Thereafter, serial sections were prepared by rotary microtome (Leitz Wetzlar, Germany) and stained with hematoxylin-eosin. To perform histomorphometric analyses, follicles were classified to preantral and antral types. Follicles with intact/complete layers of GCs and theca cells, ordinary cytoplasm of oocyte and intact nuclei were considered as normal/intact follicles. Follicles with GC dissociation, early antrum formation, luteinized elongated GCs were considered as atretic types. The atretic preantral and antral follicles were counted in serial sections for each sample and compared between groups.

### Analyses of RNA damage

Darzynkiewicz method was considered to assess the RNAdamage (23). In brief, ether alcohol was used to wash the ovaries and thereafter, 10 µm sections were obtained using cryostat microtome (Huntingdom, UK). Different degrees of ethanol were used to fix the sections. Next, the sections were rinsed in acetic acid (1%) and washed in distilled water. The slides were stained in acridine-orange (3-5 minutes) and then counterstained in phosphate buffer (pH=6.85, 2 minutes). Finally, the fluorescent colors differentiation was induced by calcium chloride. The follicular cells with RNA damage were characterized with loss and/or faint red stained RNA. The normal cells were marked with bright red fluorescent RNA.

### Immunohistochemical staining

Tissue slides were heated at 60°C (25 minutes) in a hot-air oven (Venticell, Germany). Tissue sections were then dewaxed in xylene (2 changes, each change 5 minutes) and rehydrated. Following antigen retrieval process (in 10 mM sodium citrate buffer), the immunohistochemical (IHC) staining was conducted based on the manufacturer’s protocol (Biocare, USA). Briefly, endogenous peroxidases were blocked by 0.03% hydrogen peroxide containing sodium acid. The sections were washed gently and thereafter, incubated with Erα (1:500), Erß (1:600) and c-Myc (1:500) biotinylated primary antibodies in 4oC, overnight. The slides were then rinsed gently with phosphate-buffered saline (PBS) and placed in a humidified chamber with a sufficient amount of streptavidin conjugated to horseradish peroxidase in PBS, containing an antimicrobial agent, for 15 minutes. Next, DAB chromogen was used to mark target proteins. Counterstaining was conducted by hematoxylin. Finally, the sections were dipped in ammonia (0.037 ml), rinsed in distilled water and coverslipped. The positive immunohistochemical reaction was visualized as brown.

### RNA isolation, cDNA synthesis and reverse transcription-polymerase chain reaction

Previously collected and stored (-70°C) ovaries were used for total RNA extraction, based on the standard TRIZOL method (24). In brief, 20-30 mg of ovarian tissue from individual animal of each group was homogenized in 1 ml of TRIZOL (Thermo Fisher Scientific, USA) and the colorless aqueous phase was collected. The extra care was taken in order to avoid genomic DNA contamination. The amount of total RNA was determined using nanodrop spectrophotometer (260 nm and A260/280 ratio=1.8-2.0), and thereafter the samples were stored at -70°C. For reverse transcription-polymerase chain reaction (RTPCR), cDNA was synthesized in a 20 µl reaction mixture containing 1 µg total RNA, oligo (dT) primer (1 µl), 5×reaction buffer (4 µl), RNase inhibitor (1 µl), 10 mM dNTP mix (2 µl), M-MuLV Reverse Transcriptase (1 µl) according to the manufacturer’s protocol (Fermentas, Germany). Cycling protocol for 20 µl reaction mix was performed for 5 minutes at 65°C, followed by 60 minutes at 42°C, and 5 minutes at 70°C to terminate the reaction. PCR reaction was carried out in a total volume of 27 µl containing PCR master mix (13 µl), FWD and REV specific primers (each 1 µl), and cDNA as a template (1.5 µl) and nuclease free water (10.5 µl). The PCR conditions were run as follows: one cycle of general denaturation at 95°C for 3 minutes, followed by 35 cycles of 95°C for 20 seconds, annealing temperature (50°C for *c-Myc*, 62°C for *Erα*, 58°C for Erß and finally 60°C for ß-Actin) for 60 seconds and elongation at 72°C for 1 minute, before terminating cycle at 72°C for 5 minutes (25, 26). Specific primers were designed and manufactured by Cinna-Gen company (Iran). Primers pair sequences, for individual genes are presented in Table 1. 

### Determination of ovarian TAC, MDA, SOD and GSHpx contents

In order to analyze ovarian antioxidant capacity, the tissues were washed three times with 0.9% NaCl solution, and using Teflon-end-on homogenizator (Elvenjempotter, USA), each ovary tissue was homogenized in 9 ml of 1.15% KCl. Thereafter, the homogenates were centrifuged at 4000 rpm. MDA content was next measured based on the thiobarbituric acid (TBA) reaction and the sample absorbance ratios were measured and recorded at 532 nm (27). Ovarian activities of SOD and GSH-px were analyzed using the commercial measurement kits of RAN-SOD and RAN-SOL (Rodex, Germany) and the absorbance ratio of samples were measured and recorded at 340 nm. Ovarian TAC status was also evaluated based on the ferric reducing antioxidant power (FRAP) assay and the absorbance of samples was measured and recorded at 593 nm (28). Finally, the ovarian protein contents were evaluated based on the Lowry method (29).

### Serum sampling and hormonal analyses 

Blood sample of each animal was collected directly from heart and serum was separated by centrifugation (3000 rpm for 5 minutes). Finally, serum progesterone, estrogen, testosterone, androstenedione, FSH and LH concentrations were measured. Serum levels of the hormones were evaluated by ELISA method. Moreover, intra- and inter-assay coefficient variances of the current experiment were respectively estimated as 3.1, 3.9, 4.2, 3.2 and 4.6% for testosterone, estrogen, androstenedione, LH and FSH (for 10 times), as well as 7.9, 6.3, 6.7, 7.2 and 6.3% for testosterone, estrogen, androstenedione, LH and FSH (for 10 times).

### Statistical analysis and imaging

The data were analyzed using SPSS for windows, version 16.0 (SPSS Inc., Chicago, IL, USA), presented as mean ± SD and the comparison between groups were made by analysis of variance (ANOVA) followed by Bonferroni post-hoc test. Finally, the value of P<0.05 was considered significant. SONY onboard camera (Zeiss, Cyber-Shot, Japan) was used to take photomicrographs. The pixel- based frequency for mRNA damage was analyzed using Image pro-insight software (Version 9:00, USA). 

**Table 1:** Nucleotide sequences and products size of the primers used in RT-PCR


Target genes	Primer sequence (5´-3´)	AT (˚C)	Product size (bp)

Erα	F: CCGGTCTATGGCCAGTCGAGCATC	62	380
	R: GTAGAAGGCGGGAGGGCCGGTGTC		
Erβ	F: AGCGACCCATTGCCAATCA	58	290
	R: CTGGCACAACTGCTCCCACTAA		
c-Myc	F: AACTTACAATCTGCGAGCCA	50	420
	R: AGCAGCTCGAATTTCTTCCAGATAT		
Β-Actin	F: GTTACCAGGGCTGCCTTCTC	60	310
	R: GGGTTTCCCGTTGATGACC		


RT-PCR; Reverse transcription-polymerase chain reaction and AT; Annealing temperature.

## Results

### Platelet-rich plasma diminished PCOS-induced follicular atresia and mRNA damage

**Fig.2:**
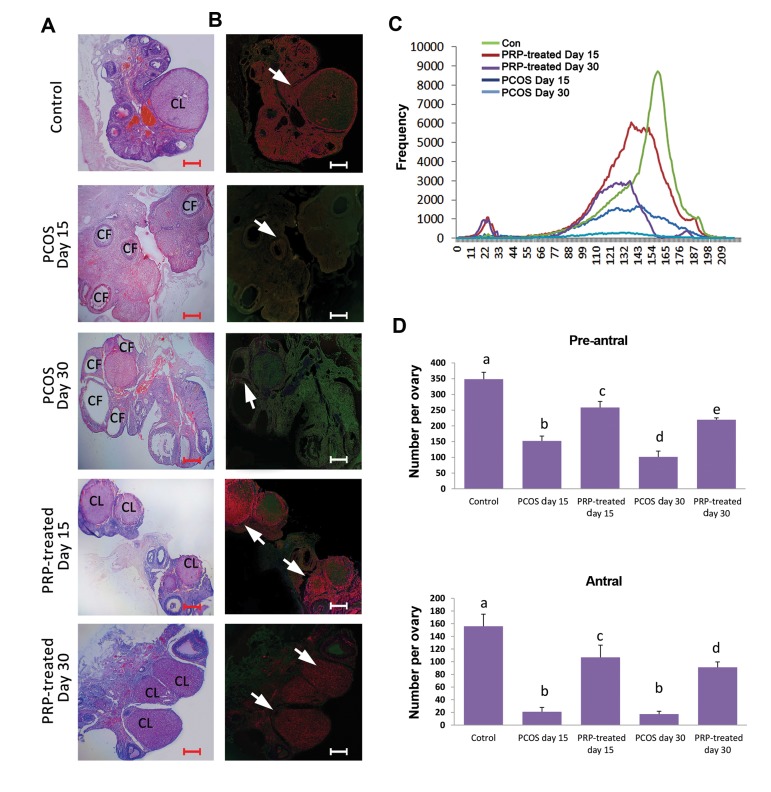
Cross sections from ovarian tissue and mRNA damage. **A.** Hematoxylin and eosin staining of ovarian cross sections in different groups; see massivecystic (CF) and atretic follicles distribution on the ovaries of the PCOS-sole groups. The ovaries from PRP-treated groups represent corpora lutea (CL) on theovaries following 15 and 30 days, **B.** Fluorescent staining for RNA damage: the cross sections of PCOS-sole groups represent damaged RNA in yellowish and/orgreen fluorescent spots (arrows). Meanwhile, the sections of PRP-treated groups exhibit intact RNA in bright red fluorescent reactions (arrows), **C. **Pixel based frequency assay for bright red fluorescent reactivity (marking intact RNA content) in 209×10 µm of tissue; see diminished reactivity in the PCOS-sole groups, and D. Mean ± SD of intact preantral and antral follicles in different groups. Different letters represent significant differences (P<0.05) between groups (n=6). PCOS; Polycystic ovarian syndrome, PRP; Platelet-rich plasm, Pre-antral: a vs. b, d, e; P=0.001, a vs. c; P=0.01, b vs. c; P=0.001, b vs. d; P=0.02, b vs. e; P=0.02, c vs. d; P=0.001, c vs. e; P=0.02, Antral: a vs. b, d; P=0.001, a vs. c; P=0.01, b vs. c; P=0.001, b vs. d; P=0.01 (scale bar: 300 µm).

Animals of the PCOS-sole groups exhibited pie size atretic/cystic follicles in the cortex of ovaries. However, the animals of PRP auto-located groups showed corpus luteom formation, representing physiologic ovulation. Histological observations showed that PRP decreased PCOS-induced follicular atresia. Accordingly, the animals of PRP auto-located groups exhibited remarkably (P<0.05) higher number of intact preantral and antral follicles/ovary versus non-treated PCOS-induced animals. Moreover, special fluorescent staining was done to assess PCOS-induced mRNA damage. The animals in PCOS-sole groups showed intensive mRNA damage. Meanwhile, those of PRP auto-located groups exhibited diminished mRNA damage in pixel based frequency analyses. No histopathological change was seen in the control group (Fig .2A-D). 

### Platelet-rich plasma enhanced Erα and Erß expression 

**Fig.3:**
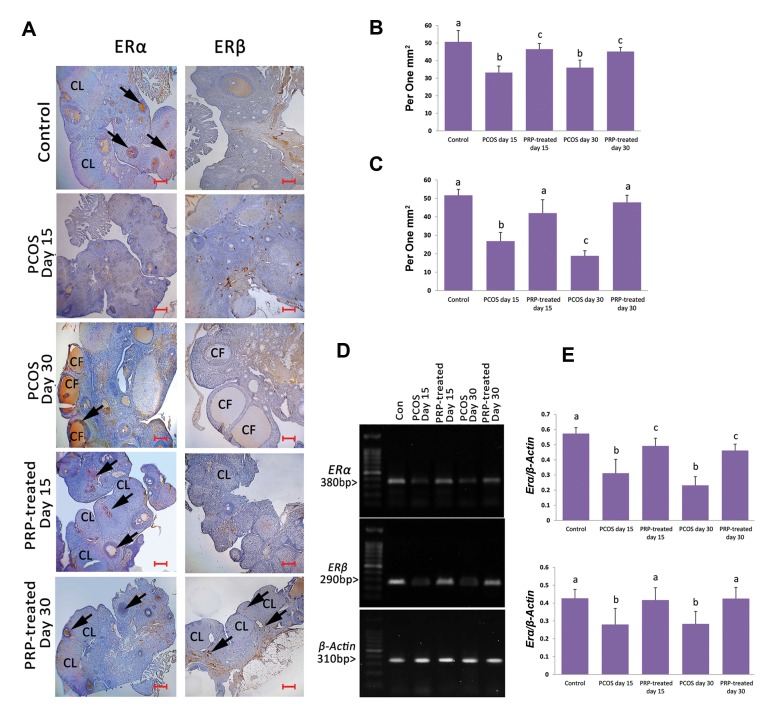
IHC staining and RT-PCR results for *Erα* and *Erβ*. **A.** See decreased Erα-positive reactions in the PCOS-sole group, while it is increased in the PRP-treated groups. Note the increased Erß-positive cells in the PRP-treated group (30 days after PCOS-induction), **B.** Mean ± SD of Erα (a vs. b; P=0.01, a vs. c; P=0.03, b vs. c; P=0.03), **C.** Erß-positive cells per 1 mm2 of tissue in different groups (n=6) (a vs. b, c; P=0.001), **D.** Electrophoresis photomicrographs of *Erα* and *Erβ* mRNA in different groups, and **E.** Density of *Erα* and *Erβ* mRNA levels in ovarian tissue, measured by densitometry and normalized to ß-Actin mRNA expression level (a vs. b; P=0.02, a vs. c; P=0.03). Arrows are representing positive reaction for *Erα* and *Erβ* antibodies. All data are represented in mean ± SD (n=6). Different letters represent significant differences (P<0.05) between groups (scale bar: 300 µm). IHC; Immunohistochemical, RT-PCR; Reverse transcription-polymerase chain reaction, ER; Estrogen receptor, PCOS; Polycystic ovarian syndrome, CF; Cystic follicle, CL; Corpus luteum, and PRP; Platelet-rich plasma.

The animals of PCOS-sole groups exhibited diminished mRNA levels of Erα and Erβ compared to the control group. However, semi-quantitative RT-PCR analyses exhibited significant (P<0.05) enhancement in mRNA levels of *Erα* and *Erβ* in the PRP auto-located groups compared to the PCOS-sole groups. More IHC analyses showed similar results, representing the elevated number of *Erα* and *Erβ*-positive cells per 1 mm2 of tissue in the PRP auto-located groups versus the PCOS-sole animals (Fig .3A-E).

### Platelet-rich plasma decreased PCOS-induced c-Myc overexpression

**Fig.4:**
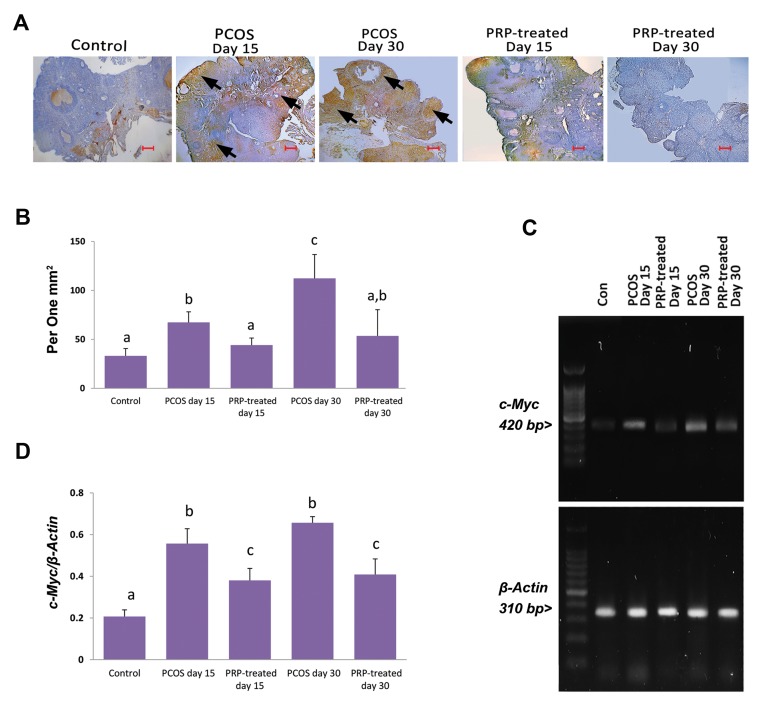
IHC staining and RT-PCR results for c-Myc. **A.** See increased c-Myc-positive cells in the PCOS-sole groups vs. the control group. PRP-treated sections represent reduced c-Myc-positive cells compared to the PCOS-sole groups, **B.** Mean ± SD of c-Myc-positive cells per 1 mm2 of tissue in different groups (n=6) (a vs. b; P=0.01, a vs. c; P=0.01, b vs. c; P=0.02), **C.** Electrophoresis photomicrographs of c-MycmRNA in different groups, and **D.** Density of c-MycmRNA levels in ovarian tissue, measured by densitometry and normalized to ß-ActinmRNA expression level (a vs. b, c; P=0.01, b vs. c; P=0.01). Arrows are representing positive reaction for c-Myc antibody. All data are represented in mean ± SD (n=6). Different letters represent significant differences (P<0.05) between groups (scale bar: 300 µm). IHC; Immunohistochemical, RT-PCR; Reverse transcription- polymerase chain reaction, PCOS; Polycystic ovarian syndrome, and PRP; Platelet-rich plasma.

The PCOS-sole animals exhibited increased expression of *c-Myc* compared to control group. Meanwhile, the animals of PRP auto-located groups showed diminished expression of *c-Myc* versus PCOS-sole groups. Accordingly, lower mRNA level and *c-Myc*-positive cells distribution were observed in PRP auto-located animals (Fig .4A-D).

### Platelet-rich plasma enhanced ovarian antioxidant status

To estimate the ovarian antioxidant potential, TAC, MDA, SOD and CSG-px levels were analyzed. Observations showed significant (P<0.05) reduction in TAC, SOD and GSH-px levels of ovaries in the PCOS- sole group versus the control animals, while, ovarian MDA content was increased in the PCOS-sole groups compared to the control group. In contrast, those animals in the PRP auto-located groups exhibited remarkable (P<0.05) reduction in MDA content and significant (P<0.05) enhancement in TAC, SOD and GSH-px levels versus the PCOS-sole group. The data for antioxidant profile are presented in Table 2. 

### Platelet-rich plasma ameliorated PCOS-induced hormonal imbalance

The PCOS-sole animal groups showed increased serum levels of FSH, LH, testosterone and androstenedione as well as diminished levels of estrogen and progesterone compared to control group. However, the animals of PRP auto-located groups exhibited diminished serum levels of FSH, LH, testosterone and androstenedione. Moreover, serum estrogen and progesterone levels were increased in the PRP auto-located groups in comparison to the PCOS- sole animals (P<0.05). The data for hormonal profile are presented in Table 3. 

**Table 2:** Serum hormone levels in different groups


Groups	Estrogen (pg/ml)	Progesterone (pg/ml)	Testosterone (ng/ml)	Androstenedione (ng/ml)	FSH (ng/ml)	LH (ng/ml)

Control	72.25 ± 19.00^a^	66.90 ± 4.33^a^	0.68 ± 0.34^a^	0.44 ± 0.07^a^	1.24 ± 0.12^a^	0.75 ± 0.10^a^
PCOS-15 D	20.33 ± 7.50^b^	12.90 ± 2.10^b^	2.21 ± 0.62^b^	0.93 ± 0.08^b^	2.45 ± 0.51^b^	1.81 ± 0.51^b^
PRP-treated 15 D	48.64 ± 5.68^c^	60.45 ± 8.91^a^	0.72 ± 0.22^a^	0.48 ± 0.13^a^	1.28 ± 0.09^a^	1.02 ± 0.26^a^
PCOS-30 D	21.37 ± 6.99^b^	10.77 ± 1.45^b^	1.67 ± 0.56^b^	1.14 ± 0.19^b^	2.84 ± 0.43^b^	1.94 ± 0.34^b^
PRP-treated 30 D	56.37 ± 3.21^c^	64.33 ± 6.41^a^	0.64 ± 0.16^a^	0.54 ± 0.10^a^	1.23 ± 0.11^a^	0.88 ± 0.10^a^


Data are presented as mean ± SD. Different letters represent significant differences (P<0.05) between data in the same row (n=6). 15 D; 15 days after PCOS-induction, 30 D; 30 days following PCOS-induction, FSH; Follicle stimulating hormone, LH; Luteinizing hormone, PCOS; Polycystic ovarian syndrome, and PRP; Platelet-rich plasma.

**Table 3:** Antioxidant profiles of ovarian tissue in different groups


Groups	TAC (mMol/mg protein)	MDA (mMol/mg protein)	SOD (U/ml)	GSH-px (U/ml)

Control	1.77 ± 0.43^a^	0.85 ± 0.10^a^	126.66 ± 7.35^a^	120.00 ± 21.01^a^
PCOS-15 D	0.45 ± 0.01^b^	2.66 ± 0.34^b^	37.66 ± 16.25^b^	63.10 ± 14.21^b^
PRP-treated 15 D	0.94 ± 0.05^c^	2.18 ± 0.06^c^	93.33 ± 7.02^c^	115.74 ± 21.37^a^
PCOS-30 D	0.59 ± 0.02^d^	3.77 ± 0.29^d^	34.00 ± 9.64^b^	51.44 ± 4.25^b^
PRP-treated 30 D	1.26 ± 0.06^e^	1.12 ± 0.150^a,c^	96.34 ± 8.34^c^	116.73 ± 14.37^a^


Data are presented as mean ± SD. Different letters represent significant differences (P<0.05) between data in the same row (n=6). 15 D; 15 days after PCOS-induction, 30 D; 30 days following PCOS-induction, TAC; Total antioxidant capacity, MDA; Malondialdehyde, SOD; Superoxide dismutase, GSH-px; Glutathione peroxidase, PCOS; Polycystic ovarian syndrome, and PRP; Platelet-rich plasma.

## Discussion

Considering cross-links between oxidative stress, hyperandrogenemia, insulin resistance and PCOS, the present study was performed to uncover the ameliorative role of PRP against PCOS-induced/related pathogenesis in animal models. Our findings showed that, auto-locating PRP significantly improved ovarian antioxidant status, down-regulated androgen synthesis and up-regulated follicular survival as well as ovulation. Moreover serum estrogen level and expression of *Erα* and *Erβ*, as important elements in follicular growth/atresia, were evaluated after PRP auto-location. Observations revealed that PRP significantly enhanced serum estrogen and progesterone levels and up-regulated ERs expression. Finally, considering the prooven pro-apoptotic role of *c-Myc* in theca interna of atretic follicles, as well as peripheral theca lutein cells, c-Myc mRNA level and *c-Myc*-positive cells distribution/1 mm2 of ovarian tissue were evaluated. The PRP auto-located groups showed a remarkable reduction in c-Myc expression versus PCOS-sole animals.

It has been well-established that in majority of cases (especially in the models with hyperandrogenemia), PCOS associates with insulin resistance and severe oxidative stress (30, 31). To understand the subject, it should be noted that hyperglycemia and higher levels of free fatty acid following insulin resistance initiate the oxidative stress by producing higher amounts of free radicals (32). On the other hand, positive correlation between oxidative stress and elevated androgen levels has been discovered in PCOS (33). Minding the androgen boosting effect of free radicals (34) as well as ameliorative effect of PRP on hyperandrogenemia and oxidative stress, serum androgen levels and ovarian antioxidant status were analyzed. Our findings showed that auto-locating PRP significantly diminished serum testosterone and androstenedione levels, improved ovarian TAC level and diminished lipid peroxidation ratio. On the other hand, any reduction in tissue levels of antioxidant enzymes, including SOD, GSH-px and catalase has been reported to initiate and promote oxidative stress in ovarian tissue (35). To show alterations, we assessed tissue levels of SOD and GSH-px. Observations revealed that PRP significantly enhanced PCOS-reduced GSH-px and SOD levels. Based on biochemical results, PRP could fairly up-regulate the ovarian GSH-px and SOD levels. Indeed, pathologically-produced oxidative stress results in severe damages at cellular levels of DNA, RNA, protein and lipid (36). Thus, we assessed RNA damage and MDA levels as biomarkers for oxidative stress-induced damages. Based on biochemical findings, PRP auto-location significantly diminished mRNA damage and reduced ovarian MDA content. Taking all these findings together, we can suggest that PRP induces antiandrogenic and antioxidant effects, at least in the case of experimentally- induced hyperandrogenic PCOS. In line with this issue and considering the boosting effect of antioxidants on meaningful follicular growth, the complementary and antioxidant chemicals are lastly used to manage/reduce the PCOS-induced pathogenesis. Consistently, various studies showed that administrating antioxidant agents are able to potentially improve insulin sensitivity and enhance the ovarian antioxidant potential in women with PCOS (37, 38). 

PCOS up-regulates serum gonadotropin levels and significantly diminishes the estrogen and progesterone synthesis versus control animals and/or fertile women (4). In corroboration with those reports, the animals in PCOS- sole groups showed higher serum LH and FSH levels, in addition to lower levels of estrogen and progesterone versus the control group. In contrast, PRP auto-location reversed the condition by reducing serum LH and FSH levels, and up-regulating estrogen and progesterone concentrations. In line with this, it has been illustrated that estrogen inflicts the GC proliferation, oocyte development, maintains the follicular survival (from atresia), promotes the ovarian angiogenesis (8, 9) and finally by binding to its nuclear receptor (*Erα* and *Erβ*) stimulates various growth factors secretion, such as *IGF* and *EGF*, resulting in follicular survival (14). However, any reduction in Erα expression results in a failed follicular maturation and/or ovulation and hemorrhagic cysts formation. In addition, the failed *Erß* expression leads to chronic anovulation (8). Thus, we can suggest that diminished estrogen secretion in PCOS- sole groups impressively inflicted follicular atresia, which ultimately resulted in an impaired ovulation. Considering significant up-regulation of follicular growth as well as diminished atresia in PRP auto-located groups, we can suggest that PRP improves follicular growth by up- regulating the estrogen secretion and enhancing the Erα and *Erß* expressions. Aside these hypotheses, it should be considered that PRP, by preserving the gonadotropins secretion, might restore the ovarian-hypophysis hormonal disruption and, by up-regulating the estrogen synthesis, promoted follicular cells proliferation and oocyte development. All of these evidences thereafter promote follicular growth and accelerate successful ovulation (marked with increased corpora lutea generation and progesterone level in PRP auto-located groups). The role of growth hormones in early (FSH-independent follicular development) and late (cell proliferation and inhibiting apoptosis) folliculogenesis should not be ignored (39). As PRP potentially contains several growth factors, it would be more logic to suggest that the ameliorative effect of PRP may partially depend on several growth hormones, which could be assessable in ovaries following PRP auto- location. 

Massive expression of c-Myc protein in GCs, theca interna of atretic follicles and peripheral theca lutein cells confirm the c-Myc-induced pro-apoptotic characteristic (11). Our RT-PCR and IHC analyses showed increased c-Myc expression in PCOS-sole groups versus control animals. However, the animal of PRP auto-located groups exhibited a diminished expression of *c-Myc*. In order to understand the subject, contrary roles of c-Myc should be highlighted. Indeed, c-Myc, under certain conditions, exerts completely opposite features. Accordingly, the estrogen (at physiologic levels) by targeting the ERs (especially *Erα*), stimulates the follicular growth through induction of G1- to S-phase transition. Actually, current induction is mainly associated with rapid and direct up-regulation of c-Myc, controlling cyclin D1 expression, cyclin-dependent kinase (CDK) activation and phosphorylation of retinoblastoma proteins (40). In contrast, c-Myc overexpression and/or inappropriate expression is sufficient to induce/promote apoptosis in GCs, theca interna of atretic follicles and peripheral theca lutein cells (10, 11). All of these evidences inflict atresia. Taking all together, we can conclude that diminished estrogen synthesis, associated with decreased expression of ERs in PCOS-sole groups, may trigger c-Myc overexpression, leading to impressive apoptosis at follicular level. However, ameliorated estrogen synthesis and up-regulated ERs expression in PRP-auto-located groups could fairly adjust the PCOS-increased c-Myc level. Diminished follicular atresia in PRP auto-located groups confirms this hypothesis. 

Although ameliorated follicular growth, enhanced ovulation ratio (marked with higher corpora lutes), up- regulated antioxidant status and balanced hormonal levels are illustrated in the current study, there are some limitations in this study -including sample size in terms of quantity, focusing on aromatization, angiogenesis and insulin resistance of animals- which should be considered in the future studies.

## Conclusion

Our preliminary data showed that auto-locating PRP fairly ameliorates PCOS-induced pathogenesis. Accordingly, it is able to suppress androgen over- synthesis and ameliorate hormonal imbalance, in addition to improvement of ovarian antioxidant status as well as inhibiting c-Myc overexpression. It can ultimately enhance ovulation ratio. Considering these findings and minding high amounts of different growth factors in PRP, auto-location of this factor could be considered as a new method for PCOS subjects. 
